# Structure, Activity and Function of the MLL2 (KMT2B) Protein Lysine Methyltransferase

**DOI:** 10.3390/life11080823

**Published:** 2021-08-12

**Authors:** Alexia Klonou, Sarantis Chlamydas, Christina Piperi

**Affiliations:** 1 Department of Biological Chemistry, Medical School, National and Kapodistrian University of Athens, 11527 Athens, Greece; alexiakl@med.uoa.gr (A.K.); schlamydas@med.uoa.gr (S.C.); 2 Research and Development Department, Active Motif, Inc., Carlsbad, CA 92008, USA

**Keywords:** MLL2, structure, H3K4me3, chromatin regulation, disease, dystonia, cancer

## Abstract

The Mixed Lineage Leukemia 2 (MLL2) protein, also known as KMT2B, belongs to the family of mammalian histone H3 lysine 4 (H3K4) methyltransferases. It is a large protein of 2715 amino acids, widely expressed in adult human tissues and a paralog of the MLL1 protein. MLL2 contains a characteristic C-terminal SET domain responsible for methyltransferase activity and forms a protein complex with WRAD (WDR5, RbBP5, ASH2L and DPY30), host cell factors 1/2 (HCF 1/2) and Menin. The MLL2 complex is responsible for H3K4 trimethylation (H3K4me3) on specific gene promoters and nearby *cis*-regulatory sites, regulating bivalent developmental genes as well as stem cell and germinal cell differentiation gene sets. Moreover, MLL2 plays a critical role in development and germ line deletions of *Mll2* have been associated with early growth retardation, neural tube defects and apoptosis that leads to embryonic death. It has also been involved in the control of voluntary movement and the pathogenesis of early stage childhood dystonia. Additionally, tumor-promoting functions of MLL2 have been detected in several cancer types, including colorectal, hepatocellular, follicular cancer and gliomas. In this review, we discuss the main structural and functional aspects of the MLL2 methyltransferase with particular emphasis on transcriptional mechanisms, gene regulation and association with diseases.

## 1. Introduction

Chromatin remodeling is a key feature of gene regulation and activity, with histone modifications playing a primary role in the modulation of the chromatin landscape and gene expression. Among the most prominent histone modifications is the methylation of histone 3 (H3) lysine (K) residues, detected on gene enhancers and specific gene promoter regions. Mono- and di-methylation of H3K4 (H3K4me1/me2) is mainly observed in enhancers whereas H3K4me3 is present on active gene promoters. Several protein lysine methyltransferases (PKMTs), including Mixed Lineage Leukemia 1-5 (MLL1-5/KMT2A-E), SET Domain-Containing 7 (SET7), SET and MYND Domain-Containing 3 (SMYD3), SET9 and PR/SET Domain 9 (PRDM9), are responsible for the transfer of methyl groups onto H3K4. The largest group of human lysine 4 (K4) HMKTs is the Mixed Lineage Leukemia (MLL/KMT2) protein family, named after the association with a subset of incurable acute leukemias of its founding member. All family members are characterized by a highly conserved catalytically active Su(var)3-9, Enhancer of zeste and Trithorax (SET) domain [1].

In yeast, there is a single MLL homolog comprised of a SET domain (SET1) which catalyzes mono-, di- and tri-methylation of histone H3K4, whereas in *Drosophila melanogaster* there are three homologs, namely, Set1, Trithorax-related (Trr) and Trithorax (Trx), responsible for H3K4 methyltransferase activity [2]. The Trithorax group of proteins has been identified as regulators of Homeotic (Homeobox) genes in *Drosophila* and are essential for body patterning in multicellular organisms. Their activity is antagonized by the Polycomb group of proteins (PcP) which exerts the repressive role in Homeobox genes expression.

Mammalian cells possess six SET1-like H3K4 methyltransferases, including the four MLL1-4 family proteins and Set1A and Set1B (KMT2F, KMT2G). Sequence homology has shown that two human homologs exist for each of the H3K4 methyltransferase proteins in *Drosophila*. More specifically, MLL1/KMT2A and MLL2 (4)/KMT2B have a similar domain structure to Trx, MLL3/KMT2C and MLL4 (2)/KMT2D are homologous to Trr while SET1A and SET1B are homologous to Set1/dSet1 [2]. Although MLL5 (KMT2E) was originally considered as an MLL family member, its divergent SET domain from the other family members as well as the lack of lysine methyltransferase activity, have re-classified it to a different subgroup of SET domain proteins.

Of importance, the MLL family members deposit distinct H3K4 methylation states and target different genomic regions. SET1A/B enzymes establish global H3K4me3 levels through a crosstalk with the monoubiquitination of the H2B process, whereas MLL1 and MLL2 catalyze the H3K4me3 modifications at specific gene promoters. MLL2 further implements H3K4me3 at bivalently marked gene promoters, while MLL3/4 enzymes mediate H3K4me1 at transcription enhancers throughout the human genome [3,4].

Although a direct functional role of H3K4 in transcription is still under investigation, the aberrant transcription mediated by MLL family members has a significant impact in gene regulation and normal cell physiology with an ultimate connection to developmental disorders and cancer [5].

Herein, we discuss the major structural and biochemical characteristics of the MLL2 (KMT2B) methyltransferase with emphasis on its cellular and molecular functions as well as its connection to diseases.

## 2. The MLL2 Protein

Genome duplication during mammalian evolution resulted in two paralogs in each MLL subgroup (MLL1/KMT2A and MLL2(4)/KMT2B) which are analogous proteins within the Trx-related subgroup, referred to as the MLX family (MLL-TRX) [6].

The *MLL2 (KMT2B)* gene (OMIM 606834) is located on chromosome 19q13.12 and consists of an 8.5–9 kb transcript, spanning 20 kb of genomic DNA. It is expressed in most human tissues [7] and has a similar genomic structure with *MLL1*, present in chromosome 11q23.

**Figure 1:**
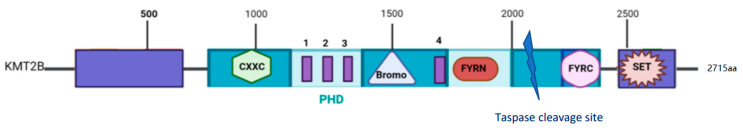
Domain structure of MLL2 (KMT2B) protein. MLL2 protein contains a CXXC domain composed of two zinc ions and four cysteine residues (CXXC), 4 plant homeotic domains (PHD) in the N-terminal region, a FY-rich N-terminal (FYRN) as well as a FY-rich C-terminal (FYRC) domain and a catalytically active C-terminal SET domain.

The MLL2 protein is 2715 amino acids in length and its structural organization includes the catalytically active C-terminal SET domain, an AT hook, a CXXC domain and several plant homeotic domains (PHD) in the N-terminal region (Figure 1) [6,8]. The SET domain forms a pocket that binds to methyltransferase cofactor S-adenosylmethionine and the N-terminal tail of histone H3 catalyzing the methylation reaction [9]. Prior to the C-terminal SET domain, the MLL2 protein displays additional structurally distinctive characteristics which determine its non-redundant role and the intrinsic biochemical and molecular functions.

The CXXC domain composed of two zinc ions and four cysteine residues (Cys4), also known as the Zinc-finger (ZF)-CXXC domain, recognizes and binds to non-methylated CpG DNA, being critical for the association of MLL2 to chromatin [10]. Both MLL1 and MLL2 contain a CXXC domain serving as a localization mechanism through the recognition of CpG islands present in most active promoters. However, MLL3/4 do not possess a CXXC domain as well as SETD1A/B which, however, are located at a complex with the CXXC domain-containing protein CFP1 which stabilizes them at promoters.

Next to the ZF-CXXC domain, MLL2 protein contains multiple PHD fingers, PHD1 to PHD4 [11] which possess a Cys4-His-Cys3 motif, coordinated by two zinc ions and mediating binding to methylated histone H3 [12]. Although all MLL family members contain PHD fingers, they exhibit different interaction specificities with the PHD3 of MLL2 being mostly involved in binding to H3K4me3 tails. Between PHD3 and PHD4, there is a bromodomain (BRD) which does not serve as a reader of acetylated lysine as commonly observed, but rather supports the PHD3 function [13]. Following the BRD, there is another PHD and a FY-rich N-terminal (FYRN) as well as a FY-rich C-terminal (FYRC) domain which allows the non-covalent dimerization of the N- and C-terminal fragments upon proteolytic cleavage [14,15,16].

MLL2 and MLL1 can be cleaved by threonine aspartase 1 (Taspase 1) [17]. Upon cleavage, the two fragments associate via a FYRN and FYRC domain interaction and form at the junction a new FYR domain [18] which has proved essential for methyltransferase activity [14,15,16]. Mice deficient in *Taspase 1* exhibit defects in cell proliferation and at the progression of the cell cycle, indicating the functional significance of the MLL2 cleavage [16].

Additional DNA-binding motifs have been detected in MLL1 and MLL2 in the form of multiple HMG-like N-terminal AT hooks which enable binding to AT-rich DNA, discriminating their binding activities from the other MLL family members [18].

In addition to the characteristic SET domain, MLL2 contains a CXXC domain followed by 4 PHD (PHD1-4), a single bromo domain (Bromo, BRD) as well as a FYRN and FYRC domain (created using BioRender).

## 3. The MLL2 Protein Complex

All MLL family proteins contain a highly conserved SET domain at their C-terminus. They all form multi-protein complexes known as COMPASS (complex of proteins associated with Set1) and COMPASS-like complexes based on their homology with Drosophila Trr, Trx and dSet1 [19,20]. These complexes share four common subunits, the so-called WRAD module.

The WRAD module regulates the enzymatic active form of the complex, confers stability and enables recruitment to chromatin. It is composed of WDR5 (WD repeat domain 5, homolog of Swd3), RbBP5 (retinoblastoma-binding protein 5, homolog of Swd1), ASH2L (absent, small or homeotic-2 like, homolog of Bre2) and DPY30 (homolog of Sdc1) subunits which are critical for H3K4 methylation activity [9,21,22,23].

**Figure 2:**
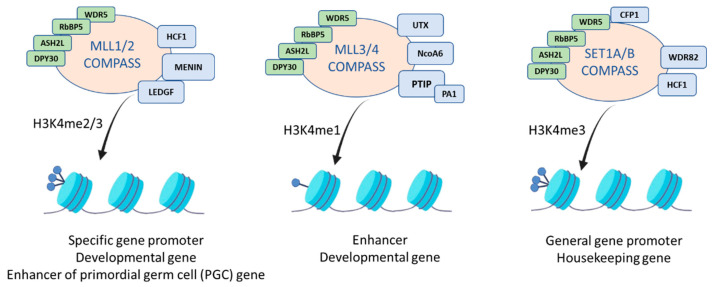
Subunit composition of the different COMPASS protein complexes showing their H3K4 methylation activities and distinctive genomic target regions.

Each COMPASS complex contains additional unique subunits on top of the main interacting proteins that enable their functional diversity (Figure 2). For MLL1/MLL2 COMPASS complexes, these proteins are Menin and host cell factors 1/2 (HCF1/2) [24] as well as the lens epithelium-derived growth factor (LEDGF), also named PSIP1/p75, which is capable of interacting indirectly with the complex through Menin.

Menin has been shown to be necessary for MLL1 target gene expression such as *Meis 1, Hoxa9, CDKN1B* and *CDKN2C*, which are required for MLL fusion protein-mediated leukemogenesis. The interaction of MLL1 and Menin forms a binding pocket for LEDGF which promotes transcriptional activation and is necessary for leukemogenesis. MLL2 shares the same interaction with Menin but not with LEDGF which is considered a unique coactivator of MLL1 complex activity. Apart of the nuclear member of the A-kinase anchoring protein family, AKAP95, MLL2 interacts only with very few interacting partners [25].

For the MLL3 and MLL4 COMPASS, the unique subunits are PTIP-associated 1 (PA1), PAX transactivation domain-interacting protein (PTIP), nuclear receptor coactivator 6 (NcoA6) and the H3K27me3 demethylase ubiquitously transcribed tetratricopeptide repeat X chromosome (UTX) [26,27,28], while the SET1A/B COMPASS complexes contain the WD repeat domain 82 (WDR82), CXXC finger protein 1 (CFP1) and HCF1 [29].

The COMPASS complexes are responsible for mono-, di- and trimethylation of H3K4 [30] and according to their unique subunits, as well as the interaction with transcriptional regulators, the COMPASS complexes exhibit a differential specificity and genome localization. Specific reader proteins can recognize H3K4 methylation and connect the relevant information underlying this modification with the basal transcription machinery; thus, enhancing transcription. Currently, a range of writers, readers and erasers that bind to methylated H3K4 via different domains [such as PHD, ZF-CXXC, tandem Tudor domain (TTD) or double chromodomains (DCD)], has been detected and is summarized in Table 1 [31,32,33].

Complex recruitment is mediated by several mechanisms to specific gene loci, either through binding to histone modifications, specific transcription factors, cofactors and long noncoding RNAs (lncRNAs). Of interest, both the core subunits as well as the complex-specific ones can interact with transcription factors to recruit the complexes to specific gene loci. Among them, Menin has been demonstrated to interact with estrogen receptor-α (ERα) and enables MLL2 recruitment to the gene locus [34]. Other transcription factors that associate with MLL complexes include the AP2δ (activating protein 2δ) [35], MYC [36], NF-E2 (nuclear factor, erythroid 2) [37], NF-Y (nuclear transcription factor Y) [38], USF1 (upstream transcription factor 1) [39], E2Fs [40], NANOG [41], PAX7 (Paired Box 7) [42] and p53 [43]. 

MLL1 has been shown to interact with transcription cofactors, including the lysine acetyltransferases MOZ, CBP and MOF, which mediate gene expression through H4K16 acetylation. Furthermore, MLL1, but not MLL2, has been demonstrated to bind to the PAF1 complex, serving as a bridge for RNA pol II, indicating a unique function of MLL1 [44]. Another distinct property of MLL1 is the interaction with repressive factors that results in negative regulation, including the PcG proteins HPC2, BMI-1 and HDAC1, c-terminal binding protein (CtBP) corepressors [45].

## 4. Structural Nucleosome Recognition by MLL Complexes

Recently, elegant single-particle cryo-electron microscopy (cryo-EM) studies have shed some light on the way MLL complexes recognize H3K4 within the nucleosome core particles (NCP).

The MLL1 complex was shown to dock on the NCP through the RbBP5 and ASH2L proteins which interact both with the nucleosomal DNA and the H4 tail. This configuration enabled the catalytic SET domain of MLL1 to align at the nucleosomal dyad and facilitate the symmetric access of H3K4 substrate to the NCP [46].

Additionally, there is evidence that the methylation of H3K4 can be induced by mono-ubiquitination of the histone H2B. A specific H2B mark on lysine 120 (H2BK120ub1) has been shown to disrupt chromatin compaction and allow the open chromatin structure. Cryo-EM studies of MLL1 and MLL3 have demonstrated their association with NCPs that contain H2BK120ub1 or unmodified H2BK120. The RBBP5 of MLL1 or MLL3 binds directly to H2B-conjugated ubiquitin. This interaction enables access to the H3 tail, which is required for H3K4 methylation. The differential organization of WDR5 and RBBP5 in MLL1 and MLL3 complexes accounts for their distinct enzymatic activities [23,47].

Another recent structural study demonstrated that the activity of the MLL family members on the NCP requires DPY30 [46]. It was shown that DPY30 interacts with the ASH2L intrinsically disordered regions (IDRs) to control MLL1 binding to NCP and regulate the complex activity. Of note, this interaction of DPY30-ASH2L-IDRs was shown to regulate all MLL family members regardless of their respective intrinsic activities. DPY30 was shown to affect global H3K4me3 levels by MLL1/MLL2, but also to mediate H3K4me1 by MLL3 in vitro. Moreover, DPY30 was essential for establishing *de novo* H3K4me3 in ESCs since its knockdown caused a global reduction in H3K4me3 [48].

Altogether, the core subunits of each MLL complex, as well as the proteins containing IDRs, exert important biophysical properties in MLL complexes and modulate their activity in chromatin.

## 5. MLL2 Role in Transcription Regulation

Several studies using Chromatin Immunoprecipitation (ChIP) followed by NGS sequencing (ChIP-seq) have shown that MLL2 can establish narrow H3K4me3 peaks at regions proximal to active gene promoters as well as co-exist with H3K27me3 in bivalent genes of embryonic stem cells (ESC). The purification of a minimal catalytical MLL2 complex (MLL2C) has demonstrated a specific methyltransferase activity for H3K4 methylation (H3K4me1/me2/me3) on recombinant histone octamers and recombinant chromatin. The stimulatory effect of the MLL2-related H3K4me on transcription has been validated using a well-established chromatin-templated in vitro transcription system [49]. Specifically, H3K4me3 at bivalent genes has been demonstrated to be mediated by the MLL2 COMPASS complex indicating an important role of MLL2 during development [50]. MLL2, but not MLL1, was shown to establish H3K4me3 on bivalent promoters in mouse ESCs (mESCs), thereby activating genes critical for the differentiation of stem cells [51,52,53,54]. Bivalent genes commonly harbor both H3K4me3 and H3K27me3 marks at the promoters in mESCs and are typically expressed only at very low levels. However, upon differentiation, they become either predominantly H3K27me3- or H3K4me3-marked and, subsequently, silenced or activated, respectively [55,56,57].

By using specific antibodies recognizing two different epitopes in the C-terminal portion of MLL2 (ab CT1 and more C-terminal ab CT2) and the ChIP-seq technique, a large number of MLL2-binding regions were identified with 70% localized to promoters, 14% to gene bodies and 16% to intergenic regions. The high occupancy of MLL2 in promoters was consistent with previous studies indicating its activity in bivalent genes. A further analysis with the ab CT1 revealed that more than 6000 MLL2 binding sites were outside of TSS, located in the intergenic and gene body regions. Moreover, the same study reported that around 39% of them shared marks with active enhancers, including p300 and H3K27ac [50]. Interestingly, a direct causal role for MLL2C-mediated H3K4 methylation was demonstrated in transcription activation and a combinatorial, synergistic effect of the p300 acetyltransferase responsible for H3K27Ac at the enhancer regions, indicating a role of MLL2 in the enhancer function in combination with histone acetylation marks [25]. Subsequently, MLL2 depletion in mESC infected with lentiviral shRNA and a ChIP-seq analysis demonstrated that MLL2 was responsible for establishing H3K4me3 marks at the non-TSS MLL2-associated genes [50].

In order to reveal the molecular mechanisms that underly MLL2 targeting to chromatin, the CRISP/Cas9 technology was used to generate MLL2 knockout mESC and was used for future rescue experiments. MLL2 was shown to bind unmethylated CpG-containing DNA indicating a possible involvement of the CXXC domain. Using several CXXC mutants, it was observed that MLL2 depends on its CXXC domain for recruitment to chromatin [50]. Through structural studies analyzing different CXXC domains in mammalian proteins, it was shown that the MLL2 CXXC domain is specific for unmethylated CpG regions in dsDNA, indicating a gene regulation role of MLL2 and a potential crosstalk with the methylation of DNA at promoters. Interestingly, CXXC domain swap experiments between MLL1 and MLL2 revealed different subnuclear localization and genomic binding patterns and, thus, a differential gene regulation [53,54]. Furthermore, it was shown that the small amino acid differences which are present around the CXXC domain of MLL2 guide it to target genes different from those of MLL1 [51]. Moreover, the CXXC domain of MLL1, but not of MLL2, associates to the Paf1/RNA Polymerase II (pol II) Complex Component (PAF1) transcription elongation complex [44,52].

Studies on MLL2 knockout mESC have demonstrated that MLL2 is not required for the expression of pluripotency genes (such as *Klf4, Oct4*), but is rather necessary for the expression of master regulators required for the primordial germ cell (PGC) specification, such as *Prdm1, 14, DDx4 and Lin28b*, during ESC differentiation [50]. Gene expression profiling and cell differentiation studies in parental MLL2 knockout and CXXC mutant mESC, demonstrated that the H3K4 methyltransferase activity of MLL2 as well as its CXXC domain are required for PGC induction [50]. Moreover, it was shown that the MLL2 COMPASS regulates the activity of enhancers and promoters of PGC gene regulators through H3K4 trimethylation, further indicating an essential role of H3K4me3 in the establishment of PGC during the differentiation of embryonic cells.

A functional interplay between H3K27me3, H3K4me3 and methylation of DNA has been detected to fine tune the expression of MLL2 gene targets in mammalian ES cells. Mechanistic studies have revealed a significant role of the MLL2 and SET1A/B complex in counteracting H3K27me3 and the methylation of DNA [49,58,59].

Of importance, a study showed that a large set of genes which exhibited increased levels of H3K27me3 upon MLL2 depletion, could be rescued by the removal of DNA methylation or the depletion of members of the PRC2 complex [58]. This overlap of the repressive mechanisms could be attributed to the potential of the H3K27me3 mark to recruit and regulate DNA methylation deposition. This hypothesis was further confirmed by demethylation with a 5-Aza-2-deoxycytidine (5dAza) treatment in mESCs (both WT and *Mll2* KO), which revealed the concomitant interplay of H3K27me3, MLL2-dependent transcription regulation and DNA methylation. Genome-scale screening indicated that the depletion of CXXC1 (component of SET1A/B complexes) in MLL2 knockout mESCs was sufficient to rescue the loss of expression of the ~1200 MLL2-dependent genes. Interestingly, the rescue of these genes expression was not correlated with the re-appearance of the H3K4me3, showing that MLL2 and H3K4me3 may have a more instructional role in gene regulation for certain types of genes, as has been reported in previous studies [60,61].

Additionally, in the vast majority of MLL2 targeted genes, the deletion of MLL2 led to the reduction in gene expression, while this repression was shown to be restored by either the removal of H3K27me3 or restoring the DNA demethylation. It was observed that there is a big overlap among genes at least partially rescued that could be explained from the fact that DNA methylation impacts H3K27me3 deposition [58]. The removal of DNMTs in triple knockout mice was further connected with global alterations of H3K27me3 levels and the dilution of the repressive effect on Polycomb-targeted genes [62].

Transcriptional kinetic studies in *Mll2*-conditional knockout mammalian ESCs have revealed the order of the events that lead to gene silencing and the crosstalk between the action of MLL2, RNA pol II processing and DNA methylation. Focusing at the *MagohB* gene, they have uncovered the mechanistic role of MLL2 in gene expression. The presence of MLL2 maintained an open chromatin state at the promoter of the target gene, regulated RNA pol II association and was correlated with active chromatin marks and high levels of mRNA. The depletion of Mll2 led to a rapid decrease in active marks (H3K4me3 and H3K9Ac) and an increase in DNA methylation at the *MagohB* gene promoter. Interestingly, DNA methylation seemed to be a secondary event to gene silencing [63].

To further evaluate the role of DNA methylation in antagonizing MLL2 in d25 GV oocytes, a study assessed the distribution of H3K4me3 in the absence of DNA methylation by using conditional knockout mice for MLL2 and double knockout for DNMT3a/b genes. It was revealed that there are two complementary, independent mechanisms of H3K4m3 trimethylation. One mechanism was transcription-dependent and was not connected to MLL2 activity, while the other relied on the specific targeting of MLL2 in unmethylated CpG-rich regions, mainly at distal elements and intergenic regions. Interestingly, these regions are protected by DNA methylation during oogenesis. The non-canonical role of MLL2 was not connected to gene expression but rather marked the bivalent chromatin state at repressed H3K27me3-marked promoters [55,64].

Additional studies on the epigenetic and expression profiling of target genes were performed to detect pathways that are regulated by MLL2 enzymatic activity and revealed mechanistic insights into the functional role of MLL2 [65]. Upon ChIP-seq and RNA-seq profiling in both wild-type and MLL2 null mammalian cells (HCT116 cells), MLL2 was found to participate in retinoic acid receptor signaling by promoting retinoic acid-responsive gene transcription. Among the genes associated with MLL2-enriched loci was the Ankyrin repeat and SOCS box protein 2 (*ASB2*) which in previous studies had been demonstrated to be induced by retinoic acid in leukemia cells. ASB2 expression in myeloid leukemia cells has been shown to induce the inhibition of proliferation and chromatin condensation. However, *MLL2^−/−^* cell lines have shown a reduction in ASB2 expression due to an effect on H3K4me3 levels [65].

The same genome-wide study demonstrated the involvement of MLL2 in different cellular pathways. Among the transcription factors that were regulated by MLL2 was the Nuclear Receptor Subfamily 3 Group C Member 1 (NR3C1) and p53. It has been demonstrated that p53 contains a sequence similar to an autoinhibitory N-terminal loop of the MLL2-SET domain. The N-terminal loop of MLL2 adopts a similar conformation as the H3 tail and, thus, enters the substrate-binding pocket of another MLL2-SET enzyme. This specific sequence and conformation of p53 makes it a perfect candidate substrate of the complex [66]. Further biochemical experiments and a mass spectrometry analysis have shown that p53 could be methylated by MLL complexes, at the K503 site, identifying a non-histone substrate for the MLL family. Preliminary experiments suggest that this newly found methylation site of p53 may affect its transcription activity and be implicated in human pathologies, including cancer [66].

## 6. MLL2 Role in Human Physiology

The *MLL2* gene was originally identified by its homology to *MLL1* and was further detected to be broadly expressed in human tissues. The ability of both paralogs to bind Menin/LEDGF, has proved critical for their normal functions [7,67].

Of importance, *Mll2* germ-line deletions have been associated with early growth retardation, neural tube defects and increased apoptosis that leads to embryonic death before E11.5 [68]. *Mll2* was involved in the preservation of the mesodermal marker Mox1 and Hoxb1 as well as in the deregulation of HoxB cluster genes. However, after E11.5, *Mll2* loss was not associated with notable pathologies indicating that it is not required for the late development and homeostasis of somatic or stem cells [68].

However, MLL2 is also implicated in germinal cell differentiation and contributes to enriched H3K4me3 marks observed in the active genes of spermatogonial stem cells. Spermatogenesis was lost upon its deletion [69]. Moreover, in oocytes global H3K4me3 mediated by MLL2 has been observed and deletions in *Mll2* resulted in anovulation and death. The elevated transcription of apoptotic factors and p53 as well as the loss of global H3K4me2/3 was also detected [70]. Additionally, it was shown that MLL2 is autonomously required for fertility and participates in epigenetic reprogramming during fertilization. However, in mid-gestation, *Mll2* deletion did not affect the global methylation of H3K4 and hematopoiesis, as observed with *Mll1* [69].

Although the majority of hematopoietic cell types do not depend on MLL2 for their function, macrophages have been demonstrated to require *MLL2* for proper cytokine signaling. Upon stimulation by lipopolysaccharide (LPS), a Rosa-CreERT2 model of *Mll2*^−/−^ macrophages from bone marrow, displayed attenuated intracellular NF-κB signaling due to reduced Toll receptor 4 (TLR4) activation. This was attributed to the loss of Phosphatidylinositol Glycan Anchor Biosynthesis Class P protein (Pigp) which adds glycophosphatidylinositol to transmembrane proteins. In turn, this induced the loss of CD14 anchoring at the cellular membrane which co-operates with TLR4 in response to LPS. Apart of the *Pigp* gene promoter, several other *Mll2* targets exhibited reduced H3K4me3 peaks in TSS and a respective increase in H3K27me3 mark which relate to repressed or bivalent genes. Therefore, MLL2 possibly maintains the expression of the target genes through H3K4me3 promoter enrichment and the resistance of invading repression complexes. However, H3K4 hypomethylated genes in *Mll2*^−/−^ macrophages exhibited no change in expression levels, indicating a higher sensitivity of some genes to H3K4me3 promoter depletion than others [71,72].

Furthermore, MLL2 is involved in cell growth control by regulating the activity of the *MYC* oncogene. MLL2 is attracted to the *MYC* enhancer by a process that involves β-catenin and promotes the transcription of *MYC* via H3K4me3 methylation [73].

## 7. MLL2 Implication in Diseases

The regulation of transcription by MLL family members is very important for human health, and mutations in *MLL* genes have been detected in several developmental disorders as well as in hematological and non-hematological cancers.

An important physiological role of MLL2 has been demonstrated in the control of voluntary movement. Specifically, MLL2 haploinsufficiency has been linked to the most severe type of a hyperkinetic movement disorder, the early onset-generalized children dystonia, which is defined by involuntary twisting postures due to sustained or intermittent contractions of agonist and antagonist muscles [5,74,75]. The patients present heterozygous mutations in the *MLL2* gene and characteristic brain magnetic resonance imaging findings with a typical facial appearance and possible progress to cranial and laryngeal dystonia over time [76].

Gene expression profiling in patients harboring *MLL2* mutations has shown that certain proteins associated with dystonia, such as torsin family 1 member A (TOR1A), THAP domain-containing, apoptosis-associated protein 1 (THAP1) and dopamine receptor D2 (D2R) are decreased in cerebrospinal fluid and fibroblasts, indicating MLL2 implication in disease pathogenesis that needs further investigation [5].

Moreover, in adult mice, conditional *MLL2* deletion in excitatory forebrain neurons resulted in learning impairment due to increased activity of genes involved in hippocampal plasticity via H3K4me2/3 [50,76].

Another disease-promoting role has been attributed to MLL2 in respect to cell proliferation enhancement and carcinogenesis [31]. As originally identified, somatic mutations of *MLL1* have been associated with cancer onset. The *MLL1* gene exhibits a considerable number of rearrangements with several other translocation partner genes, possibly attributed to the inability of developing hematopoietic cells to repair the frequent chromosomal double-strand DNA breaks [31]. The MLL1-fusion proteins are coded by exons 8-13 forming the C-terminal part of the hybrid protein and a variable number of fusion partner exons coding the N-terminal. Upon the translocation of *MLL1* to its fusion partners, the H3K4 methyltransferase activity is lost due to the loss of the SET domain. More than 135 *MLL1* rearrangements have been identified up to date, being mostly in frame translocations that lead to the generation of gain-of-function oncoproteins with altered activities [77]. The fusion of translocation gene partners results in the formation of complexes which may interact with other methyltransferases such as the disruptor of telomeric silencing 1-like (DOT1L) to induce H3K79 methylation and alter gene expression in favor of a leukemic transformation.

It is interesting to note that although MLL2 exhibits a structural similarity to MLL1, it is not related to chromosomal translocations and exhibits a lower affinity for DNA binding at unmethylated CpG sequences, being unable to replace MLL1 in leukemic oncoproteins [68,77,78]. Several common genes can be fused with *MLL1*, including MLL-ENL, MLL-ELL, MLL-AF4, MLL-AF9, MLL-AF10 and MLL-PTD, accounting for 80–90% of MLLs, whereas MLL1-rearranged leukemias account for 10% of all leukemias [31,77].

In MLL1-rearranged leukemias (MLL-AF9), deletion of the *MLL2* gene (wt) was shown capable of decreasing the leukemic cell survival, but WT-*MLL1* deletion had no impact on leukemia cell function, since targeting the N-terminal part, that is shared in the MLL1-fusion protein, did ablate leukemia cells [79]; thus, indicating that the activities of the two genes are not redundant, as previously suggested [31,80].

Of interest, conditional or germline *Mll2* mutations in mice were not capable of inducing carcinogenesis [67,68]. However, *MLL2* mutations detected in cancers are mostly nonsense, missense or frameshift, and mainly involve the PHD and SET domains [6]. Mutation rates are higher in uterine corpus endometrial carcinoma (UCEC), esophageal sarcomatoid carcinoma and in gastric cancer [81,82,83]. Additionally, somatic mutations of *MLL2* have been detected in neurofibromatosis 1-glioblastoma (NF1-GBM), leading to the truncation of the MLL2 protein and have been associated with early steps of gliomagenesis [84].

The overexpression of MLL2 has also been detected in pancreatic cancer cells and additional translocations have been observed in glioblastomas [85].

In colorectal cancer, MLL2 has been reported to promote cell proliferation through physical interaction with β-catenin which allows the recruitment of MLL2 to the enhancer element of *c-MYC,* inducing its transcription [73]. MLL2 target genes, profiling in both wild-type and MLL2 null mammalian colon cancer cells (HCT116 cells), revealed that MLL2 promotes retinoic acid-responsive gene transcription such as *ASB2* which was previously induced in leukemia cells. Other transcription factors that were regulated by MLL2 include NR3C1 and p53, explaining the potential mechanistic implication of MLL2 in cancer progression [66].

Additionally, MLL2 has been revealed in genomic studies as a recurrent target for the integration on oncogenic viruses (hepatitis B virus and adeno-associated virus type 2) of hepatocellular carcinoma (HCC) tissues [86,87], indicating a potential relationship of elevated MLL2 expression with liver cancer progression that needs to be further investigated.

Furthermore, in follicular lymphoma (FL), *MLL2* mutations were frequently detected at a similar rate to t(14;18) translocation which is the molecular hallmark of the disease, indicating a central role of MLL2 in tumorigenesis [88].

Finally, in squamous-cell cancer of the head and neck (SCCHN), somatic mutations of *MLL2* were frequently detected at a 17.9% mutation rate [89]. Since these mutations were inactivating, it is suggested that MLL2 has a tumor-suppressor role in head and neck cancer, potentially changing the expression of global gene sets.

## 8. Conclusions

Taken together, all the significant progress that has occurred in recent years in understanding chromatin accessibility mechanisms and their role in gene regulation, H3K4me1/me2/me3-enriched genomic regions were demonstrated to be of primary importance. Furthermore, structural and functional studies of the MLL methyltransferase family in mediating these histone marks in specific tissues have revealed unique, non-redundant functions despite the similarity between paralogs.

MLL2 is particularly significant in mediating H3K4me3 in specific promoters of development-related genes, but is also required for H3K4me3 accumulation on bivalent promoters in ES cells. Moreover, an extensive range of H3K4 methylation-reader domains has been detected in many transcriptional coactivators demonstrating the direct stimulatory effects of the MLL complex-mediated H3K4 methylation on transcription. Therefore, studies determining the factors that enable the recruitment of MLL1/2 complexes to specific loci in the genome are highly demanded. MLL2 plays multiple and significant roles in the regulation of physiological voluntary movement; it is involved in childhood dystonia and in the pathogenesis of several malignancies. It is, thus, important to determine how to target MLL2 with small molecule inhibitors in different settings. Current efforts are directed to the development of inhibitors that target H3K4 methyltransferase activities or MLL1/2-associated subunit interactions in controlling the H3K4 methyltransferase function of MLL complexes. Major efforts were focused on the identification of chemicals that treat leukemias caused by MLL1 rearrangements. Two molecules are currently in phase I/II clinical trials (NCT04065399, NCT04067336) for Menin–MLL inhibition (SNDX-5613 from Syndax Pharmaceuticals and KO-539 from Kura Oncology) for MLL-rearranged leukemias which show promising results. Selected inhibitors can either act on proteins recruited to the MLL1 complex that are required to maintain the leukemic state or block the methyltransferase activity of MLL1 by interrupting its interaction with WDR5, Menin or LEDGF [90]. Other approaches include the direct inhibition of MLL1 activity, associated metabolic pathways and protein degradation or, alternatively, the inhibitory targeting of the BRD4 domain recruited to the *MYC* gene, switching-off *MYC*-dependent leukemia [90,91]. Importantly, the core subunits of MLL complexes are frequently amplified in different cancer types, exhibiting an oncogenic role and, therefore, present potential targets for cancer patients that need to be further explored [31].

Furthermore, recent experimental evidence suggests that MLL-associated transcriptional regulatory mechanisms, independent of the H3K4 methyltransferase activities of the complexes, are also involved in gene regulation and need to be taken into consideration as well as further investigated in functional studies.

## Figures and Tables

**Table 1:** H3K4 writers, readers and erasers.

Writers	Readers	Erasers
MLL1 MLL2 MLL3 MLL4 SET1A SET1B	BPTF (Bromodomain PHD Finger Transcription Factor) INGs (inhibitor of growth) RAG2 (Recombination Activating 2) TAF3 (TATA-Box Binding Protein Associated Factor 3) CHD1 (Chromodomain Helicase DNA Binding Protein 1)	JARID1A-D (Lysine-specific demethylase 5A, KDM5A) LSD1 (Lysine-specific histone demethylase 1A)/KDM1A (me1/2) JMJD2A (Jumonji domain-containing 2A)/KDM4A (Lysine Demethylase 4A) LSD1/KDM1B (me1/2)

## Data Availability

Not applicable.
